# The length of stay and inpatient burden in inpatients with different psoriasis subtypes

**DOI:** 10.7150/ijms.67083

**Published:** 2022-09-06

**Authors:** Qiaolin Wang, Liping Jin, Kun Hu, Minjia Tan, Yan Lu, Yingchao Zhao, Tingyin Chen, Mingliang Chen, Wu Zhu, Yehong Kuang

**Affiliations:** 1 Department of Dermatology, Xiangya Hospital, Central South University, Changsha, China 410008.; 2 National Clinical Research Center for Geriatric Disorders (Xiangya Hospital), Changsha, China 410008.; 3 Hunan Engineering Research Center of Skin Health and Disease; Hunan Key Laboratory of Skin Cancer and Psoriasis (Xiangya Hospital), Changsha, China 410008.; 4 Hospital for Skin Diseases, Institute of Dermatology, Chinese Academy of Medical Sciences & Peking Union Medical College, Nanjing, China 210042.; 5 Department of Information, Xiangya Hospital, Central South University, Changsha, China 410008.

**Keywords:** inpatient, length of stay, burden, cost of care, psoriasis, psoriatic arthritis

## Abstract

**Background:** Heavy disease burden of psoriasis has been indicated by previous studies. However, the cost of care and length of stay (LOS) in inpatients with different psoriasis subtypes were rarely addressed. This study aimed to investigate the cost of care and LOS in Chinese patients with different psoriasis types and to clarify the independent factors affecting LOS.

**Methods:** We conducted a cross-sectional study by enrolling patients with psoriasis who were hospitalized between 13 Feb 2017 and 29 Mar 2021. Demographic and clinical characteristics of the patients were collected by reviewing their Electronic Medical Records. Multivariate linear regression was used to estimate the associations with adjustments.

**Results:** A total of 310 adult patients with psoriasis were included (mean cost of care: 13.0±22.3 kCNY; mean LOS: 7.9±4.3 days). Statistically significant differences were found among patients with different psoriasis subtypes in LOS (P<0.001) but not in the cost of care (P=0.530). Relative to psoriasis vulgaris, pustular psoriasis (Adjusted coefficient: 2.37, 95% confidence interval (CI): 0.87-3.87) and erythrodermic psoriasis (Adjusted coefficient: 2.92, 95%CI: 1.38-4.47) were significantly associated with an increased LOS. Meanwhile, respiratory tract infections (Adjusted coefficient: 1.60, 95%CI: 0.11-3.10) also significantly increased the LOS. On the contrary, a decreased LOS was found in psoriatic arthritis patients treated with TNF-alpha inhibitors (Adjusted coefficient: -2.21, 95%CI: -4.37 to -0.05).

**Conclusions:** LOS differed significantly among different psoriasis subtypes while the inpatient burden for a single hospitalization was alike. Infection is an important factor associated with a longer LOS. TNF-alpha inhibitors evidently reduced the total hospital stay period for patients with psoriatic arthritis.

## Introduction

Psoriasis is a chronic inflammatory disorder with various manifestations involving skin, joints and enthesis, and can potentially affect multiple organ systems 1, 2. Previous systematic reviews reported a prevalence of psoriasis ranging from 0.91% to 8.5% in adult patients, with over 125 million people being affected globally 3-5. In China, a prevalence of 0.47% of psoriasis has been reported in 2012 6, implying a significantly increased trend in comparison with 0.12% reported in 1987 7. Psoriasis has a significant negative impact on the quality of life of patients owing to skin and joint function damage, and psychological impairment, etc. Published data has demonstrated associations between psoriasis and other disorders, such as metabolic diseases, infections, cardiovascular diseases, and psychological diseases 8-11. Inpatient care is important for patients with moderate to severe psoriasis, and heavy disease burden of psoriasis has been indicated by previous studies owing to the disease itself or its comorbidities 12-15. However, the cost of care and length of stay (LOS) in inpatients with different psoriasis types including psoriasis vulgaris (PsV), psoriatic arthritis (PsA), pustular psoriasis (PP) and erythrodermic psoriasis (EP) were rarely addressed.

In the present study, we aimed to investigate the cost of care and LOS in patients with different psoriasis types and to clarify the independent factors affecting LOS, so as to provide new implications for the management of psoriasis in clinical practice.

## Methods

### Study design and participants

As a cross-sectional study, we consecutively enrolled 310 inpatients with psoriatic diseases who sought medical consultation in Xiangya Hospital between 13 Feb 2017 and 29 Mar 2021. All patients visited the outpatient clinic of dermatology firstly, and moderate to severe psoriasis patients who needed systemic examination and treatment would be advised for inpatient care. The patients included in our study were diagnosed by experienced dermatologists (Professor Wu Zhu and Yehong Kuang). Diagnosis of PsA was based on the Criteria of the Classification of Psoriatic Arthritis 16, and dermatoscope and skin biopsy were used for patients who were difficult to diagnose. Patients aged below 18 were excluded. This study was implemented following the Declaration of Helsinki and was approved by the institutional review board at Xiangya Hospital.

### Data collection

Demographic and clinical characteristics of the patients were collected by reviewing their Electronic Medical Records. The data extracted included age, sex, educational level (primary/middle school, high school, and college or above), metabolic diseases (hypertension, dyslipidemia, diabetes, hyperuricemia), cigarette smoking and alcohol drinking, psoriasis duration, biologics use during inpatient care (TNF-alpha inhibitors, IL-17 inhibitors), infections during inpatient care, Dermatology Life Quality Index (DLQI), cost of care and LOS. Cigarette smoking was defined as having smoked at least 100 cigarettes in one's lifetime, and alcohol drinking was defined as consumption of 30g of alcohol per week for at least one year. Cost of care was calculated based on the total charge for a single hospitalization.

### Statistical Analysis

Continuous variables with normal distribution were expressed as mean ± standard deviation (SD), and were compared by analysis of variance (ANOVA). Categorical variables were summarized as counts (percentages), and were compared using the Fisher's exact test or chi-square test. Multivariate linear regression was used to estimate the associations with adjustments. To determine the predictors associated with an increased LOS, multivariate linear regression modeling was constructed with LOS as the dependent variable and clinical and demographic information as independent variables, and stepwise regression was conducted. All the data was analyzed with R version 4.0.4. P<0.05 was considered statistically significant.

## Results

A total of 310 adult patients with psoriasis were enrolled in the present study. Demographic and clinical characteristics of the patients are presented in Table 1 (mean age: 48.5±14.0 years; female: 32.2%; mean cost of care: 13.0±22.3 kCNY; mean LOS: 7.9±4.3 days). Sex, cigarette smoking, psoriasis duration, biologics use, and LOS were significantly different among the four psoriasis types.

**Figure 1:**
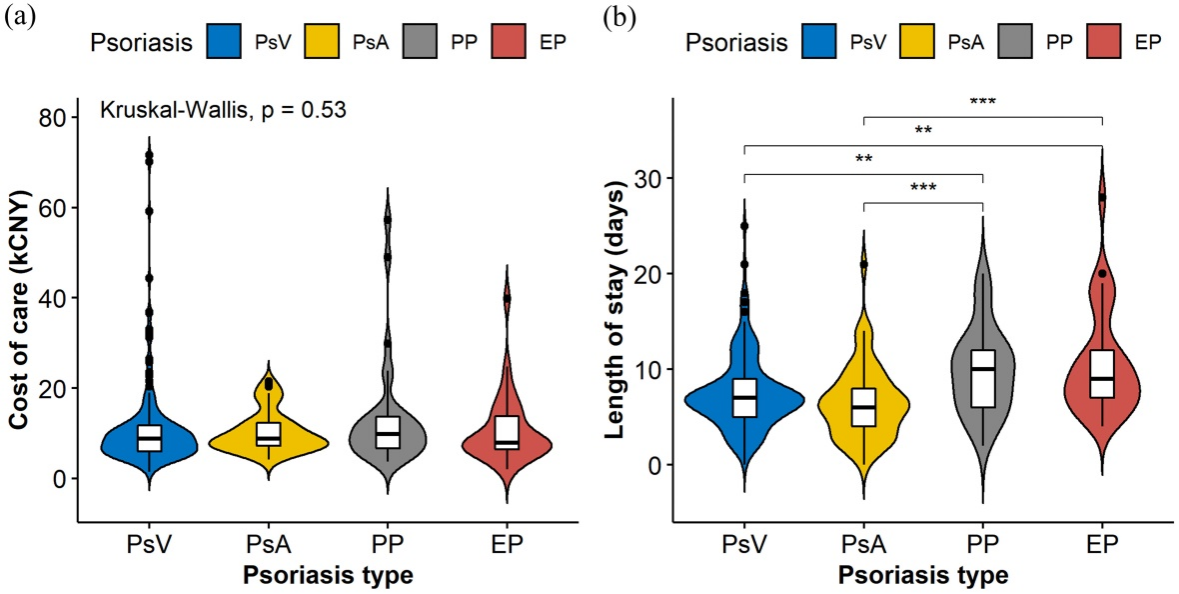
The violin plots were constructed to display the distributions of cost of care (**a**) and length of stay (**b**) in different psoriasis types. The plots indicate the smoothed densities of each distribution, and boxplots indicating medians and upper and lower quartiles. Asterisks show statistically significant differences with P values <0.01 (**) and <0.001 (***).

**Figure 2:**
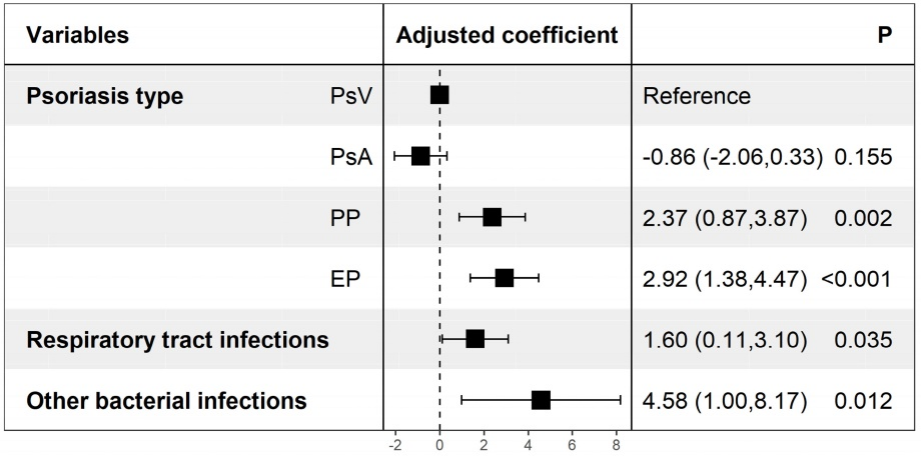
Factors associated with increased length of stay. Adjusted coefficients were yielded from multivariate linear regression modeling, and stepwise regression was conducted. Other bacterial infections including urinary tract infection, bacteremia, digestive tract infection and liver abscess. PsV: psoriasis vulgaris; PsA: psoriatic arthritis; PP: pustular psoriasis; EP: erythrodermic psoriasis.

Violin plots (Figure 1) were constructed to illustrate the distributions of the cost of care and LOS in different psoriasis types, which indicate statistically significant differences among different psoriasis types in LOS (*P<*0.001) but not in the cost of care (*P*=0.530). Table 2 shows the possible factors that are associated with an increased LOS indicated by univariate linear regression modeling. To further identify the independent factors, multivariate linear regression modeling with stepwise regression was conducted (Figure 2), which implied that PP (Adjusted coefficient: 2.37, 95% confidence interval (CI): 0.87-3.87) and EP (Adjusted coefficient: 2.92, 95%CI: 1.38-4.47) were significantly associated with an increased LOS compared with PsV (Figure 2). In addition, respiratory tract infections (Adjusted coefficient: 1.60, 95%CI: 0.11-3.10) and other bacterial infections (Adjusted coefficient: 4.58, 95%CI: 1.00-8.17) also significantly increased the LOS. Biologics were mostly used in PsV and PsA. A decreased LOS was found in PsA patients treated with TNF-alpha inhibitors (Adjusted coefficient: -2.21, 95%CI: -4.37 to -0.05) other than IL-17 inhibitors (*P*=0.221), but not in PsV patients treated with biologics (*P*>0.05).

## Discussion

In this study, we found that LOS differed significantly among the four psoriasis types while the inpatient burden was alike. PP and EP were evidently associated with an increased LOS compared with PsV and PsA. Meanwhile, infections of the respiratory tract also significantly increased the LOS. Our study also indicated that TNF-alpha inhibitors evidently reduced the total hospital stay period for patients with PsA while IL-17 inhibitors did not.

Psoriasis was considered a systemic inflammatory disease. According to previous studies, not only the psoriasis itself but also its comorbidities obviously increased the economic burden of psoriasis 17, and LOS was significantly prolonged for patients with psoriasis compared with those without psoriasis 14. It was reported that up to 30% of the patients with psoriasis might develop PsA, and PsA could further worsen the quality of life of patients owing to progressive joint damage and increased cardiovascular risk, resulting in a heavier disease burden 12, 18. In addition, nail lesions were common in PsA, with a prevalence of 41-93% (the prevalence was relatively low in psoriasis alone, ranging from 15% to 50%) 19-21. The nail involvement linked to a higher disease burden 22. By contrast, our study focused on a single hospitalization record, and found that the total cost of inpatient care was similar among the four psoriasis subtypes while PP and EP were evidently associated with an increased LOS compared with PsV and PsA. This suggested that a longer time was needed to improve the skin lesions in PP and EP, and availability of highly efficient therapies, such as biological treatment, might make quick improvement of PsV/PsA to shorten the period of hospitalization. Hsu et al. 14 reported that the mean LOS in US inpatients with psoriasis was 5.4±0.2 days, and an increased LOS linking to the Asian race was found, which was consistent with our result that the mean LOS in the Chinese inpatients was 7.9±4.3 days. In addition, the mean cost of care in the study by Hsu et al. 14 was 7433±254 USD, which was significantly higher than our data in the Chinese population. Conway et al. 23 reported that the mean LOS in UK inpatients with psoriasis was 16.8 days (median 15; IQR 8-23) and the mean inpatient cost was about £2681 while the data needed to be updated. Readmission for inpatient care is common in psoriasis in view of that it is a recrudescent chronic inflammatory disorder. Published data showed that more than one third of the inpatients with psoriasis were readmitted within one year, and the readmission was associated with a longer LOS and more infectious complications 24. Moreover, about two thirds of the total inpatient costs of psoriasis inpatients were caused by readmission 25. The economic burden of a longer time range among different psoriasis subtypes should be further examined.

In our data, the mean LOS of all psoriasis inpatients was 7.9 days, and infections of the respiratory tract significantly increased the LOS. Similar results that infections could cause a longer LOS have been reported by previous studies 9, 26. The emergence of biologics has greatly improved the quality of life for patients with psoriasis. Our study indicated that TNF-alpha inhibitors evidently reduced the total hospital stay period for patients with PsA while IL-17 inhibitors did not. TNF-alpha inhibitors were recommended with high priority for PsA, especially for patients with predominantly axial involvement and less skin lesions 27. However, no markedly shorter LOS was observed in PsV patients treated with biologics in our data. Further studies with bigger sample size and more comprehensive data of psoriasis severity should be conducted to assess the associations.

This study has several limitations. First, due to the nature of a single center study, the generalizability of our findings was limited. Second, psoriasis severity, as an important factor linking to disease burden and LOS, was not addressed in our study. Third, more confounders associated with LOS should be analyzed.

In conclusion, we found that LOS differed significantly among the four psoriasis types while the inpatient burden was alike for a single hospitalization. PP and EP were evidently associated with an increased LOS compared with PsV and PsA, and infection was also an important factor associated with a longer LOS. In addition, TNF-alpha inhibitors evidently reduced the total hospital stay period for patients with PsA. The cost of care and LOS related to readmission among different psoriasis subtypes should be further examined. Since psoriasis patients generally suffer from a heavy disease burden, continuous effort should be paid in searching effective and low-cost therapies for psoriasis.

## Figures and Tables

**Table 1:** Demographic and clinical characteristics of the patients

Characteristics	All patients (n=310)	Psoriasis type				
		PsV (n=188)	PsA (n=58)	PP (n=33)	EP (n=31)	*P*
Age (mean±SD)	48.5±14.0	47.5±14.9	48.6±11.0	50.3±13.6	51.9±13.9	0.365
Female, n (%)	100 (32.2)	52 (27.7)	24 (41.4)	16 (48.5)	8 (25.8)	0.035
**Education, n (%)**						0.307
Primary/middle school	140 (45.2)	79 (42.0)	24 (41.4)	17 (51.5)	20 (64.5)	
High school	80 (25.8)	49 (26.1)	16 (27.6)	9 (27.3)	6 (19.4)	
College or above	90 (29.0)	60 (31.9)	18 (31.0)	7 (21.2)	5 (16.1)	
**Metabolic diseases, n (%)**						
Hypertension	69 (22.3)	34 (18.1)	17 (29.3)	10 (30.3)	8 (25.8)	0.170
Dyslipidemia	67 (21.6)	41 (21.8)	14 (24.1)	4 (12.1)	8 (25.8)	0.513
Diabetes	51 (16.5)	31 (16.5)	10 (17.2)	7 (21.2)	3 (9.7)	0.658
Hyperuricemia	25 (8.1)	13 (6.9)	4 (6.9)	4 (12.1)	4 (12.9)	0.541
Cigarette smoking, n (%)	146 (47.1)	99 (52.7)	17 (29.3)	12 (36.4)	18 (58.1)	0.005
Alcohol drinking, n (%)	101 (32.6)	68 (36.2)	13 (22.4)	10 (30.3)	10 (32.3)	0.271
Psoriasis duration (mean±SD)	11.1±10.1	10.8±9.7	10.8±10.1	8.8±9.5	16.4±11.6	0.015
**Biologics use, n (%)**						<0.001
No	275 (88.7)	175 (93.1)	38 (65.5)	31 (93.9)	31 (100)	
TNF-alpha inhibitors	27 (8.7)	10 (5.3)	15 (25.9)	2 (6.1)	0 (0)	
IL-17 inhibitors	8 (2.6)	3 (1.6)	5 (8.6)	0 (0)	0 (0)	
**Infections, n (%)**						
No infections	199 (64.2)	127 (67.6)	34 (58.6)	21 (63.6)	17 (54.8)	
Latent tuberculosis	44 (14.2)	24 (12.8)	11 (19.0)	4 (12.1)	5 (16.1)	0.657
Respiratory tract	32 (10.3)	15 (8.0)	6 (10.3)	5 (15.2)	6 (19.4)	0.197
Hepatitis B virus	33 (10.6)	20 (10.6)	8 (13.8)	3 (9.1)	2 (6.5)	0.738
Skin and soft tissue	7 (2.3)	2 (1.1)	1 (1.7)	2 (6.1)	2 (6.5)	0.061
Other bacterial infections	5 (1.6)	4 (2.1)	0 (0)	0 (0)	1 (3.2)	0.468
Other viral infections	8 (2.6)	3 (1.6)	3 (5.2)	1 (3.0)	1 (3.2)	0.290
DLQI (mean±SD)	8.5±6.0	8.0±5.5	8.8±6.0	8.6±7.3	11.2±6.8	0.076
Cost of care (kCNY) (mean±SD)	13.0±22.3	14.2±27.9	10.2±4.5	13.3±11.8	10.7±7.5	0.530
Cost of care (USD) (mean±SD)	2039.1±3495.3	2224.1±4372.4	1604.9±699.7	2082.8±1848.9	1683.2±1175.2	0.650
Length of stay (days) (mean±SD)	7.9±4.3	7.5±3.8	6.5±3.9	9.8±4.5	10.6±5.5	<0.001

Other bacterial infections including urinary tract infection (n=2), bacteremia (n=1), digestive tract infection (n=1) and liver abscess (n=1). Other viral infections including herpes zoster virus infection (n=4), hepatitis C virus (n=3) and HIV infection (n=1). PsV: psoriasis vulgaris; PsA: psoriatic arthritis; PP: pustular psoriasis; EP: erythrodermic psoriasis. CI: confidence interval. TNF: tumor-necrosis factor. DLQI: Dermatology Life Quality Index.

**Table 2:** Factors associated with increased length of stay: results from univariate analysis

Variables	Univariate analysis	
	Unadjusted coefficient (95% CI)	*P*
Age	-0.003 (-0.037, 0.031)	0.868
Female	-0.26 (-1.28, 0.76)	0.618
**Education**		
Primary/middle school	ref	
High school	-1.2 (-2.35, -0.02)	0.046
College or above	-1.0 (-2.12, 0.13)	0.083
**Psoriasis type**		
PsV	ref	
PsA	-0.92 (-2.13, 0.28)	0.133
PP	2.39 (0.88, 3.91)	0.002
EP	3.16 (1.60, 4.71)	<0.001
**Metabolic diseases**		
Hypertension	0.73 (-0.41, 1.87)	0.211
Dyslipidemia	0.30 (-0.86, 1.46)	0.611
Diabetes	0.10 (-1.18, 1.39)	0.874
Hyperuricemia	-0.84 (-2.59, 0.90)	0.343
Cigarette smoking	-0.05 (-1.00, 0.90)	0.919
Alcohol drinking	-0.14 (-1.15, 0.88)	0.791
Psoriasis duration	-0.02 (-0.07, 0.02)	0.355
**Biologics use**		
No	ref	
TNF-alpha inhibitors	-2.16 (-3.83, -0.48)	0.012
IL-17 inhibitors	-0.17 (-3.15, 2.81)	0.910
**Infections**		
Latent tuberculosis	0.80 (-0.56, 2.17)	0.246
Respiratory tract	1.94 (0.39, 3.49)	0.014
Hepatitis B virus	-0.04 (-1.58, 1.50)	0.958
Skin and soft tissue	0.44 (-2.76, 3.64)	0.787
Other bacterial infections	4.62 (0.88, 3.62)	0.016
Other viral infections	1.30 (-1.69, 4.30)	0.393
DLQI	0.06 (-0.02, 0.14)	0.145

Unadjusted coefficient and P value were yielded from univariate linear regression modeling. PsV: psoriasis vulgaris; PsA: psoriatic arthritis; PP: pustular psoriasis; EP: erythrodermic psoriasis. CI: confidence interval. TNF: tumor-necrosis factor. DLQI: Dermatology Life Quality Index.

## Abbreviations

LOS: length of stay
PsV: psoriasis vulgaris
PsA: psoriatic arthritis
PP: pustular psoriasis
EP: erythrodermic psoriasis
DLQI: Dermatology Life Quality Index
SD: standard deviation
CI: confidence interval
